# The Underlying Mechanisms: How Hypothyroidism Affects the Formation of Common Bile Duct Stones—A Review

**DOI:** 10.1155/2012/102825

**Published:** 2012-09-19

**Authors:** Johanna Laukkarinen, Juhani Sand, Isto Nordback

**Affiliations:** Department of Gastroenterology and Alimentary Tract Surgery, Tampere University Hospital, Teiskontie 35, FIN-33521 Tampere, Finland

## Abstract

For decades, one well-known risk factor for the development of gallbladder stones has been hypothyroidism. Recent studies have interestingly reported that the risk in particular for common bile duct (CBD) stones increases in clinical and subclinical hypothyroidism. There are multiple factors that may contribute to the formation and/or accumulation of CBD stones in hypothyroid patients, including decreased liver cholesterol metabolism, diminished bile secretion, and reduced sphincter of Oddi relaxation. This paper focuses on the mechanisms possibly underlying the association between hypothyroidism and CBD stones. The authors conclude that when treating patients with CBD stones or microlithiasis, clinicians should be aware of the possible hypothyroid background.

## 1. Introduction

Several factors affecting bile content and bile flow are involved in the complex pathogenesis of gallstones. In hypothyroidism, not only the risk for gallbladder stones [1, 2], but also the risk for common bile duct (CBD) stones in particular is increased [3–5]. Impaired liver cholesterol metabolism [6], diminished bile secretion [7], and reduced sphincter of Oddi (SO) relaxation [8, 9] may contribute to the formation and/or accumulation of CBD stones in hypothyroid patients. In this paper the possible mechanisms underlying the association between hypothyroidism and CBD stones are being discussed.

## 2. Review Criteria

PubMed was searched in April 2011 with the terms “hypothyroidism,” “subclinical hypothyroidism,” “thyroxine,” “thyroid function,” “gallstones,” “bile duct stones,” “sphincter of Oddi,” “biliary motility,” “cholesterol metabolism,” and “hepatic secretion” for full-length English language original publications and review articles published between 1950 and 2010 (initial inclusion criteria). Abstracts and articles not relevant to the topic were excluded. A total of 3472 publications were identified at the initial step, out of which 3396 were excluded and 76 were finally considered. The search was updated in August 2012.

## 3. Prevalence of Clinical and Subclinical Hypothyroidism in CBD Stone Patients

Several recent studies report an association between hypothyroidism, or subclinical hypothyroidism, and CBD stones (Table 1). In a retrospective study on patients over 60 years of age [3], it was noted for the first time that CBD stone patients have significantly more diagnosed hypothyroidism (11%), not only when compared to control patients from whom gallstones had been excluded (2%), but also when compared to gallbladder stone patients without CBD stones (6%). In this study, there was no difference between groups in the frequency of other diseases. This finding suggested that factors other than merely those affecting cholesterol metabolism, for example, specific effects on bile flow, might be behind the association between CBD stones and hypothyroidism.

A prospective study [4] showed that even subclinical hypothyroidism is more common among CBD stone patients. This study investigated the prevalence of previously undiagnosed subclinical hypothyroidism in clinically euthyreotic CBD stone patients compared to nongallstone controls. It was found that 5.3% of the CBD stone patients had subclinical hypothyroidism, defined as serum thyrotropin above the normal upper limit (6.0 mU/L), compared to only 1.4% in the control group. In women over 60 years, the prevalence of subclinical hypothyroidism was as high as 11.4% in the CBD stone group compared to 1.8% among the control patients.

Finally in 2010, a large, medical registry-based study from Finland [5] confirmed that hypothyroid patients did indeed seem to have a higher likelihood for CBD stone treatment. In this study, the prevalence of CBD stone treatments was investigated in patients with diagnosed hypothyroidism and compared to age, sex, and area of residence adjusted glaucoma (control) patients. Patients with other diseases were excluded to create a “purely” hypothyroid (or glaucoma) cohort of patients. Out of 14,334 patients in each group who met the inclusion criteria, 0.23% in the hypothyroid cohort and 0.16% in the control cohort had been treated for CBD stones. The groups did not differ in the number of CBD stone treatments before the diagnosis of hypothyroidism or glaucoma, but after these diagnoses there were 56% more CBD stone treated individuals in the hypothyroid cohort than in the control cohort. This may suggest that the higher risk for CBD stones in hypothyroid patients may increase after taking medication for hypothyroidism. As the process of bile stone formation takes time, stone formation may have started during the untreated period of hypothyroidism and have been completed regardless of thyroxine replacement therapy. This hypothesis is supported by the findings that both subclinical [4] and clinical [3] hypothyroidism are more common in CBD stone patients. However, the question remains whether thyroxine replacement therapy is sufficient to cause the physiological effects of thyroxine, as it seems that even though thyroxine replacement therapy has been initiated, the CBD stones do indeed form or continue to grow. Earlier studies with subclinical hypothyroid patients have demonstrated that a positive effect on changes in the cholesterol level, cardiovascular effects, or neuromuscular symptoms may be achieved with early replacement treatment with thyroxine [10, 11]. It has also been reported that gallstones have dissolved after initiation of thyroxine therapy [12]. It is possible that thyroxine replacement therapy is not sufficient, or not sufficient at all times of the day, in all patients to maintain normal sphincter of Oddi function, causing the formation of CBD stones. 

These interesting findings raise a question about the mechanisms underlying the association, which is stronger between hypothyroidism and CBD stones than between hypothyroidism and gallbladder stones.

## 4. How Hypothyroidism May Affect the Formation of CBD Stones?

In general, the pathogenesis of gallstones is a complex process involving mechanisms affecting bile content and bile flow. There are several factors that may contribute to the formation of CBD stones in hypothyroid patients. Based on the investigations currently available, it cannot be concluded whether hypothyroid individuals develop isolated CBD stones or present with CBD stones in addition to gallbladder stones. However, based on what is known about the effects of hypothyroidism on the formation of gallbladder and CBD stones, it seems likely that in hypothyroidism both the risk for gallbladder-originated as well as for de novo CBD stones is increased. In hypothyroidism, the lack of thyroxine (1) decreases liver cholesterol metabolism [6] resulting in bile cholesterol supersaturation, which in turn impairs the motility [13], contractility [14], and filling [15] of the gallbladder, contributing to the retention of cholesterol crystals and to the nucleation and growth of gallstones [13]; (2) diminishes bile secretion from hepatocytes [7] resulting in impaired clearance of precipitates from the bile ducts; (3) reduces SO relaxation [8, 9] resulting in delayed bile flow [16, 17] and thus the formation and accumulation of CBD stones.

THs regulate multiple functions in virtually every type of vertebrate tissue [18, 19]. Most actions of THs can be explained by their interaction with nuclear receptors, which are expressed in a tissue- and development stage-specific fashion [20–24]. In human, the SO expresses both TR *β*_1_ and *β*_2_ [9]. Any acute-type response of a cell to THs is unlikely to involve a transcriptional mechanism but is rather a result of nongenomic mechanisms involving extranuclear sites of action [25–35]. In general, thyroid hormone actions are largely intracellular events that require transport across the plasma membrane. Recently, several active and specific thyroid hormone transporters have been identified, including monocarboxylate transporter 8 (MCT8), MCT10, and organic anion transporting polypeptide 1C1 (OATP1C1) [36, 37].

### 4.1. Hypothyroidism Decreases Liver Cholesterol Metabolism

A 90% of hypothyroid patients have elevated cholesterol levels, triglyceride levels, or both [38–42]. Treatment of hypothyroid patients with concomitant hyperlipidemia will have beneficial effects on serum cholesterol levels [40]. In hypothyroidism, decreased LDL receptor activity leads to impaired removal of cholesterol from the serum [38, 43, 44], and reduced regulation of HMG-CoA reductase expression leads to decreased cholesterol synthesis [45, 46]. Even though THs reduce the synthesis of bile salts in human hepatocytes [46], a decrease in biliary bile salt concentration in hypothyroidism has been reported [47]. Hypothyroidism lowers biliary cholesterol secretion in rat, while thyroxine replacement in hypothyroid animals markedly increases cholesterol secretion [48]. However, in cholesterol-fed hypothyroid rat, biliary cholesterol content is significantly increased and the rate of bile secretion decreased [7]. 

Serum hypercholesterolemia in hypothyroidism may cause bile to supersaturate in cholesterol. A direct consequence of cholesterol supersaturated bile is reduced motility [13], depressed contractility [14], and impaired filling [15] of the gallbladder, giving rise to prolonged residence of bile in the gallbladder. This may contribute to the retention of cholesterol crystals, thereby allowing sufficient time for nucleation and continuous growth into mature gallstones [1, 3]. In one prospective study [17], biliary transabdominal ultrasonography was performed in patients with gallbladder *in situ* in the euthyreotic phase, and then again 2–4 weeks after thyroidectomy in the hypothyroid stage. In these ultrasonography studies no gallstones, sludge, or dilatation of the bile ducts were seen in the hypothyroid stage compared to the euthyreotic stage. Thus, the 2–4 week period of hypothyroidism following thyroidectomy was not long enough to cause the formation of such cholesterol crystals which could be detected in transcutaneous ultrasonography; a longer period of time is needed. Gallbladder fasting volume and gallbladder ejection fraction measured by conventional ultrasonography have also been reported to remain unchanged between euthyreotic, hypothyroid, and hyperthyroid patients [49]. In one patient report, gallstones were shown to disappear after thyroxine treatment [12]. Extremely high doses of thyroxine have also been reported to induce gallbladder stones in hamsters [50], but this has not been reported with physiological doses of thyroxine.

### 4.2. Hypothyroidism May Reduce Hepatic Bile Secretion

In a prospective study in humans, the dynamic Tc^99 m^ HIDA biligraphy performed in the acute hypothyroid stage after thyroidectomy showed that the hepatic maximal uptake and appearance of radioactivity in the large bile ducts at the hepatic hilum was similar to the euthyreotic stage in the same patients [17]. This suggested that hepatocytic bile secretion may not be significantly reduced in humans in the early phase of hypothyroidism. However, in rats, where bile secretion rate can be measured by cannulating bile ducts proximal to the SO (to block out the SO effect), decreased bile secretion in prolonged hypothyroidism has been reported, whereas hyperthyroidism seems to have no effect [7, 51]. Thus decreased bile hepatic secretion may have at least some impact on the delayed bile flow in prolonged hypothyroidism. 

### 4.3. Hypothyroidism Reduces Bile Flow into the Duodenum

In a rat study where the effect of SO was not excluded by cannulation, hypothyroidism reduced and hyperthyroidism increased the bile flow into the duodenum [16]. Similarly, in a prospective human study [17], hepatic clearance was significantly decreased and the hilum-duodenum transit time had a tendency to increase in the hypothyroid stage after thyroidectomy, when compared to the euthyreotic stage in the same patients. As the hepatic maximal uptake and the appearance of radioactivity in the large bile ducts at the hepatic hilum were similar in the hypothyroid and euthyreotic stages of this study, the findings are hardly attributable to different hepatic secretion but strongly suggest that bile flow into the duodenum is reduced in the hypothyroid stage [17]. This could be due to changes in bile composition and gallbladder motility, and because of changes in the resistance to flow, that is, in the SO motility.

### 4.4. Hypothyroidism Leads to Impaired SO Relaxation

The existence of gastrointestinal hypoactivity in hypothyroidism has been well known for decades [52–58]. For example, the effect of thyroxine has been documented in anal canal pressure and in lower esophageal sphincter pressure [59, 60]. The effect of THs on smooth muscle contraction depends on the smooth muscle type and the species studied. THs have a direct, relaxing effect on vascular smooth muscle contractility [31, 61, 62]. This effect is mediated by intranuclear binding of TH to the TR [61, 63, 64], and partly by nongenomic mechanisms involving extranuclear sites of action [25]. The potassium (K^+^) channel blocker glibenclamide attenuates triiodothyronine-induced vasodilatation in rat skeletal muscle arteries, and triiodothyronine-induced vasodilatation may thus be mediated by ATP-sensitive K^+^-channels [65].

Since Sandblom et al. [66] first demonstrated the hormonal action of cholecystokinin (CCK) on the SO in 1935, several other hormones have been shown to affect SO activity [67–71]. In 2001 [8], it was shown for the first time that thyroxine has a direct effect on SO contractility in physiological concentrations in pig experiments. Triiodothyronine had a similar effect on thyroxine, whereas cortisone, estrogen and testosterone had no effect. Thus the effect of THs is not an unspecific effect of any hormone. Progesterone, which is thought to be involved in the smooth muscle relaxation seen in pregnancy [72], reduced not only the ACh- and Hist-induced but also KCl-induced SO contractions. Thus, its effect on SO relaxation differs from the more specific effect of thyroxine. Thyroxine reduced receptor-mediated acetylcholine and histamine-induced SO contraction, but had no effect on unspecific, KCl-induced SO contraction, which suggests a direct effect of thyroxine on the control mechanisms of SO motility. Since the effect of thyroxine on the precontracted SO is relaxing, the absence/insufficient concentration of thyroxine may result in increased tension of the SO in hypothyroidism [9]. A similar relaxant effect of thyroxine was also shown in human SO specimens, indicating that the finding may also be of clinical significance [9].

## 5. Mechanisms by Which Thyroxine Mediates SO Relaxation

Several examinations were performed to determine how the relaxant effect of thyroxine on SO is mediated [9]. The experiments with *α*- and *β*-adrenoceptor antagonists, NO-synthesis inhibitor, and the elimination of nerve function with tetrodotoxin showed that the thyroxine-induced relaxation of SO is not mediated via neural effects. Human SO was shown to express TR *β*_1_ and *β*_2_. The presence of TRs [9] in the SO is necessary but not sufficient evidence that thyroxine exerts its prorelaxant effect via a hormone-receptor complex action. However, the experiments with different incubation times of thyroxine showed that the underlying cellular mechanisms involved do not act immediately but require a certain time lag, supporting the theory that at least part of the action of thyroxine is TH-TR mediated [9]. The passage of TH through cell membrane, cytoplasm, and nuclear membrane and binding to a nuclear protein (TR) is a relatively fast event, whereas the resulting transcriptional and translational regulation is time-consuming, and probably explains why the relaxant effect is not immediate. Thus, the effect of thyroxine could be mediated by regulatory proteins partly synthesized as a result of thyroxine-induced gene expression.

Estrogen is one hormone known to affect the synthesis prostaglandins [73], which, in turn, can relax smooth muscle [74]. However, in prostaglandin synthesis inhibition experiments it was shown that prostaglandin synthesis is not required in the mediation of the prorelaxant effect of thyroxine. 

K^+^ channels can be modulated by neurotransmitters and other messengers in smooth muscle cells, and these effects are often functionally important in the whole tissue [75]. Similarly to what has been shown in rat skeletal muscle arteries, where triiodothyronine-induced vasodilatation is mediated by ATP-sensitive K^+^ channels [65], it was shown in SO that the effect of thyroxine on SO smooth muscle is mediated via the opening of ATP-sensitive K^+^ channels [9]. This results in hyperpolarisation, which closes all membrane Ca^2+^ channels and reduces Ca^2+^ influx, allowing only limited contraction of the smooth muscle [76]. 

The prorelaxant effect of thyroxine is probably partly mediated via transporter proteins [36, 37], and partly via binding to nuclear receptors, subsequently leading to the activation of K^+^ channels [9]. The opening of K^+^ channels is followed by hyperpolarisation, which closes cell membrane Ca^2+^ channels, reduces Ca^2+^ influx, and results in reduced contraction of the SO smooth-muscle cell in response to any specific stimulus.

## 6. Conclusions and Clinical Implications

In summary, several recent studies report an association between hypothyroidism, or subclinical hypothyroidism, and CBD stones. The higher prevalence of hypothyroidism in CBD stone patients compared to gallbladder stone patients suggests that not only changes in the cholesterol metabolism, or bile excretion rate, but particularly changes in the function of the SO that may underline the association between CBD stones and hypothyroidism. It remains to be investigated whether hypothyroid individuals who have had their gallbladder removed are at an increased risk to develop CBD stones when compared to euthyroid individuals in the same situation.

It seems likely that the lack of thyroxine in hypothyroidism gives rise to a reduction in bile flow in many ways. In addition to the increased cholesterol load in bile and the reduced bile secretion rate, the deficiency of the prorelaxant effect of thyroxine on the SO appears to be a crucial factor leading to the reduced bile flow in hypothyroidism.

The initial formation of bile cholesterol crystals may begin during the untreated period of hypothyroidism, and the stones may continue to develop or mature even after the thyroxine replacement therapy has begun. It is possible that thyroxine replacement therapy is not sufficient in all patients to maintain normal SO function, causing increased risk of CBD stone formation. Studies with subclinical hypothyroid patients have demonstrated that a positive effect on the changes in the serum cholesterol level, on cardiovascular effects, or on neuromuscular symptoms may be achieved with early replacement treatment with thyroxine [10, 11], and it can be assumed that patients at risk of forming CBD stones due to subclinical hypothyroidism may also benefit from such early treatment. Most importantly, when treating patients with CBD stones or microlithiasis, clinicians should be aware of the possible hypothyroid background and consider examining the thyroid function, at least in female patients over 60 years of age, in which group the prevalence of clinical and subclinical hypothyroidism is the highest.

## Figures and Tables

**Table 1:** Data of the studies published over the last ten years, reporting an association between hypothyroidism, or subclinical hypothyroidism, and CBD stones.

Author	Year	Journal	Study type	Patient population	Patient number	Gender	Age (years)	Main finding	Risk factors other than hypothyroidism contributing to development of CBD stones
Inkinen et al. [8]	2001	Hepato-gastroenterol	Retrospective	CBD stone patients, age and sex matched GB stone patients and controls	168	65% F	>60	Hypothyroidism: CBD 11%, GB 6%, controls 2%	Groups did not differ in any other diagnosed diseases

Laukkarinen et al. [4]	2007	J. Clin. Endocrinol. Metab.	Prospective, multicenter	Clinically euthyreotic CBD stone patients and nongallstone controls	445	61% F	median 67 (range 18–98)	Subclinical hypothyroidism (TSH > 6.0 mU/L): CBD 5.3%, control 1.4%.	Groups did not differ in any other diagnosed diseases
					161	F	>60	Subclinical hypothyroidism: CBD 11.4%, control 1.8%	Groups did not differ in any other diagnosed diseases

Laukkarinen et al. [5]	2010	Scand. J. Gastroenterol.	Medical registry-based cohort	Hypothyroid patients and age, sex, and area of residence adjusted glaucoma (control) patients	28.668	68% F	median 62 (range 18–88)	CBD stone treatments: hypothyroid cohort 0.23%, control cohort 0.16%. After diagnosing hypothyroidism 56% more CBD stone treatments than after diagnosing glaucoma.	Patients with other diseases were excluded from the cohort to create a “purely” hypothyroid (or glaucoma) cohort of patients. Also no difference in any other medications between the groups.
